# Prevalence and Clinical Profile of Celiac Disease in Yemeni Children: A Five-Year Retrospective Study at Al-Sabeen Hospital

**DOI:** 10.7759/cureus.82824

**Published:** 2025-04-23

**Authors:** Shafa A Alsofi, Nasher A Alaghbari, Najla N Al-Sonboli, Haitham M Jowah

**Affiliations:** 1 Department of Pediatrics, Faculty of Medicine and Health Sciences, Sana’a University, Sana'a, YEM; 2 Department of Pediatrics, Al-Sabeen Maternity and Child Hospital, Sana'a, YEM; 3 Department of Pediatric Surgery, Faculty of Medicine and Health Sciences, Sana’a University, Sana'a, YEM

**Keywords:** celiac disease, clinical manifestations, feeding practices, pediatric malnutrition, prevalence, risk factors, vaccination, yemen

## Abstract

Introduction: Celiac disease (CD) is an autoimmune condition triggered by gluten ingestion. Limited data are available on its prevalence and characteristics in Yemen, a region facing socioeconomic challenges intensified by conflict. This study aimed to estimate the prevalence of CD and evaluate the demographic, clinical, and nutritional profiles of affected children.

Methods: This five-year retrospective study analyzed data from 120 children diagnosed with CD at Al-Sabeen Hospital, Sana’a, Yemen, from January 2018 to December 2023. Children of any age and sex clinically suspected of having CD based on gastrointestinal (e.g., chronic diarrhea) and/or extraintestinal manifestations (e.g., failure to thrive) were included, with non-CD causes excluded via European Society for Pediatric Gastroenterology, Hepatology, and Nutrition (ESPGHAN)-guided testing. Diagnosis followed the guidelines of the ESPGHAN guidelines, using transglutaminase 2 antibody (tTA-IgA) and endomysial antibody IgA (EMA IgA) levels, with biopsy recommended for tTA-IgA <10× the upper limit of normal. Data on demographics, nutritional status, clinical manifestations, and associations were collected via a structured questionnaire and analyzed using IBM SPSS Statistics for Windows, Version 26.0 (Released 2019; IBM Corp., Armonk, New York, United States), with chi-square tests assessing significance (p<0.05).

Results: Among 3,570 admissions, CD prevalence was 3.4% (n=120), with a female predominance (58.3%, n=70) and 70% (n=84) diagnosed before the age of one year (mean 12 ± 3.5 months). Malnutrition affected 60.0% of cases, significantly associated with rural residency (p=0.015), low family income (p=0.001), unprotected water sources (p=0.030), and incomplete vaccination (p<0.001). Chronic diarrhea (85.0%) and pallor (81.7%) were the most common manifestations. No significant associations were found for sex (p=0.705) or animal contact (p=0.053).

Conclusions: CD prevalence in Yemeni children exceeds the global average, with malnutrition being a major comorbidity linked to socioeconomic and environmental factors. Targeted screening, biopsy-confirmed diagnosis for ambiguous cases, and nutritional interventions are critical in conflict-affected settings such as Yemen. Future multicenter studies with genetic testing are recommended to enhance our understanding and management.

## Introduction

Celiac disease (CD) is an autoimmune disorder with a genetic basis that primarily targets the small intestine and is characterized by an inappropriate immune response to gluten, a protein found in wheat, rye, and barley. This immune reaction leads to inflammation and damage to the intestinal mucosa, impairing nutrient absorption and resulting in various deficiencies and a broad spectrum of clinical manifestations [1]. Gee first documented the symptoms of CD in children in 1887, describing signs such as irritability, chronic diarrhea, and failure to thrive, and recommended dietary changes as a treatment [2]. Since Gee’s initial observations, our understanding of CD has expanded substantially, particularly regarding its epidemiology, diagnosis, and management [3]. Globally, CD affects approximately 1% of the population; however, it often remains undiagnosed, leaving many individuals unaware of their condition [4,5].

CD can present at any age, with diagnostic peaks during early childhood and between the ages of 35 and 55 years [6,7]. Notably, CD is more frequently diagnosed in females than males, a trend that reverses in adults aged ≥ 60 years [8,9]. Genetic factors significantly influenced CD risk, with a positive family history of increased susceptibility [3]. The primary treatment for CD involves strict, lifelong adherence to a gluten-free diet (GFD), which effectively reduces the symptoms and promotes mucosal healing. However, managing a GFD can be challenging because of the risk of accidental gluten exposure and potential nutrient deficiencies. Gluten-free products are often less nutritious and more expensive than their gluten-containing equivalents [7].

The prevalence of CD varies considerably in the Arab region, with studies reporting rates ranging from 0.14% in Tunisia to 3.2% in Saudi Arabia [10]. Specific high-risk populations, such as the Saharawi in Algeria, exhibit prevalence rates as high as 5.6%, likely due to genetic factors, such as high consanguinity rates and specific genetic markers [11]. In Middle Eastern countries such as Iran, Turkey, Palestine, and Syria, the prevalence of CD is similar to that in Western countries, which is attributed to a combination of genetic predispositions and dietary patterns [12]. Despite these insights, a pronounced gap exists in the CD data from certain Arab regions, including Yemen, highlighting the need for localized studies.

Yemen presents a unique setting for studying CD because of the country’s limited healthcare infrastructure and socioeconomic challenges, which are further intensified by the ongoing conflict. Access to diagnostic resources and treatment is often constrained, potentially leading to underdiagnosis and inadequate CD management, particularly among children. The pediatric population is of particular interest because untreated children with CD can experience long-term consequences, including malnutrition, stunted growth, and developmental delays. Given the high rates of poverty, malnutrition, and limited access to healthcare in Yemen, children with CD face a compound risk of complications associated with malabsorption and immune response dysfunction. Additionally, environmental factors such as poor sanitation and inadequate access to clean water may further exacerbate the health outcomes of children with diarrhea [5,12].

This study addresses the gap in regional data by examining the prevalence, demographic characteristics, and clinical profiles of children diagnosed with CD at the Al-Sabeen Hospital in Sana’a City. Given Yemen’s unique challenges, such as limited healthcare infrastructure and high rates of malnutrition exacerbated by ongoing conflict, this study aimed to provide critical insights to guide targeted interventions and improve pediatric health outcomes in a high-need setting. Ultimately, the findings can inform healthcare strategies and contribute to the development of effective management approaches tailored to resource-limited regions, such as Yemen and beyond.

## Materials and methods

This retrospective, cross-sectional, hospital-based study was conducted from January 2018 to December 2023 at the Al-Sabeen Hospital for Maternity and Childhood in Sana’a, Yemen. It assessed the prevalence and clinical characteristics of CD among pediatric patients admitted to Al-Sabeen Hospital, Sana'a, Yemen. This study design enabled a descriptive analysis of the demographic and clinical data, offering insights into CD presentation and associated factors within the study population. As a primary referral center, Al-Sabeen Hospital serves a diverse population from both urban and rural regions, making it an ideal site for examining the prevalence and characteristics of pediatric CD cases in the region.

Participants

The study included children of any age and sex admitted to Al-Sabeen Hospital during the study period, clinically suspected of having CD based on gastrointestinal symptoms (e.g., chronic diarrhea) and/or extraintestinal manifestations (e.g., failure to thrive, pallor), as assessed by clinical evaluation. Non-CD causes (e.g., infectious diarrhea and inflammatory bowel disease) were determined through clinical assessment and diagnostic testing per the European Society for Pediatric Gastroenterology, Hepatology, and Nutrition (ESPGHAN) guidelines [13], and such patients were excluded, ensuring specificity for CD.

CD diagnostic criteria

CD diagnosis in this study was performed following the updated 2020 guidelines of the ESPGHAN [13]. Total immunoglobulin A (IgA) and IgA antibodies against tissue transglutaminase (tTA-IgA) were measured as the initial tests. The diagnosis was made without biopsy in cases where tTA-IgA levels exceeded 10 times the upper limit of normal (ULN) and were confirmed by positive endomysial antibody IgA (EMA IgA) in the second sample. Biopsy was recommended for patients with lower tTA-IgA levels, consistent with the ESPGHAN guidelines [13] and supported by Hill et al. [14], who emphasize biopsy confirmation in ambiguous cases. Additionally, we used the ESPGHAN online diagnostic tool [15] to ensure consistent, guideline-aligned diagnoses for all patients.

Variables

The primary dependent variable in this study was the prevalence of CD. Secondary dependent variables included nutritional status and frequent clinical manifestations in children diagnosed with CD. Nutritional status was assessed using WHO growth standards, with malnutrition defined as weight-for-age or height-for-age below -2 SD. Independent variables included age, sex, residence (urban or rural), water supply source, family income, contact with animals, number of household members, vaccination history, nutritional history, and past and family history of autoimmune conditions. Associations between nutritional status and independent variables were analyzed to understand the factors that exacerbate malnutrition in patients with CD.

Data collection

Data were collected using a structured questionnaire (see Appendices), developed based on a literature review and the study objectives. The questionnaire included sections on personal information, such as age, gender, residency, source of water supply, economic status, contact with animals, number of household members, body measurements (weight and height), family history of chronic diarrhea, vaccination history, and feeding history. The study also assessed gastrointestinal and extraintestinal manifestations as well as past and family history of autoimmune conditions.

Sample size

The sample size was calculated using the Epi Info software (Centers for Disease Control and Prevention, Atlanta, Georgia, United States) [16], assuming a 6.5% CD prevalence according to a previous study [17], with 95% confidence and 80% power, yielding 93 cases. To account for a 25% potential non-response rate, the final sample size was adjusted to 120 cases, consistent with the standard practice in retrospective studies [10].

Statistical methods

Data analysis was performed using IBM SPSS Statistics for Windows, Version 26.0 (Released 2019; IBM Corp., Armonk, New York, United States). Descriptive statistics were used for nominal and categorical variables, reported as frequencies and percentages, whereas quantitative data were summarized as means and standard deviations. Associations between nutritional status and other variables were assessed using the chi-square test with a significance level of p<0.05.

Ethical considerations

This study was approved by the Ethics Committee of the Faculty of Medicine and Health Sciences, Sana’a University (approval number: 517). Written informed consent was obtained from each child’s parent or caregiver before data collection, providing clear explanations about the study’s aims, confidentiality protocols, and the right to withdraw at any time. We anonymized and securely stored all data to ensure privacy and restricted access to authorized research personnel. We rigorously maintained data confidentiality in accordance with the Declaration of Helsinki and the local regulations governing research in conflict zones.

## Results

Demographic characteristics

**Figure 1:**
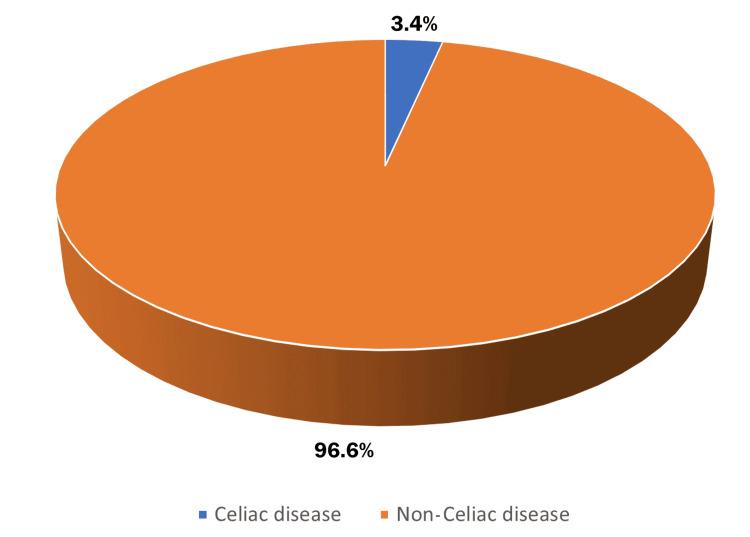
Prevalence of celiac disease

The prevalence of CD among children admitted to the Department of Children’s Nutrition at Al-Sabeen Hospital from 2018 to 2023 was 3.4%, with 120 out of 3,570 admissions diagnosed with CD (Figure 1).

Among the diagnosed cases, female predominance was observed (58.3%, n=70) compared with male (41.7%, n=50). The mean patient age was 12 ± 3.5 months, with 70% (n=84) diagnosed before 12 months of age. Most children resided in rural areas (65.0%, n=78) and were from low-income families (70.8%, n=85), with a high proportion (57.5%, n=69) living in households with five or more persons. Contact with animals was reported in 48.3% (n=58) of cases, and 55.8% (n=67) used unprotected water sources, potentially reflecting rural environmental exposures, though associations with malnutrition were not significant (p=0.053 and p=0.030, respectively). Notably, 72.5% (n=87) of mothers had no education or low education, and 15% (n=18) of cases reported a positive family history of diarrhea (Table 1).

**Table 1:** Demographic characteristics of the study population (N=120) Descriptive statistics were applied to present the demographic data. Categorical variables are expressed as frequencies and percentages, while continuous variables are summarized with the mean ± standard deviation (SD).

Variable	Frequency	Percentage
Sex		
Male	50	41.7%
Female	70	58.3%
Age (mean ± SD)	12 ± 3.5 months	
<1 year	84	70.0%
1–2 years	21	17.5%
2–5 years	15	12.5%
Residence		
Rural	78	65.0%
Urban	42	35.0%
Contact with animals		
Yes	58	48.3%
No	62	51.7%
Water source		
Protected	53	44.2%
Unprotected	67	55.8%
Family income		
Low	85	70.8%
Middle	31	25.8%
High	4	3.3%
Household size		
<5 persons	51	42.5%
≥5 persons	69	57.5%
Maternal education		
None or low	87	72.5%
Secondary or higher	33	27.5%
Family history of diarrhea		
Positive	18	15.0%
Negative	102	85.0%

**Figure 2:**
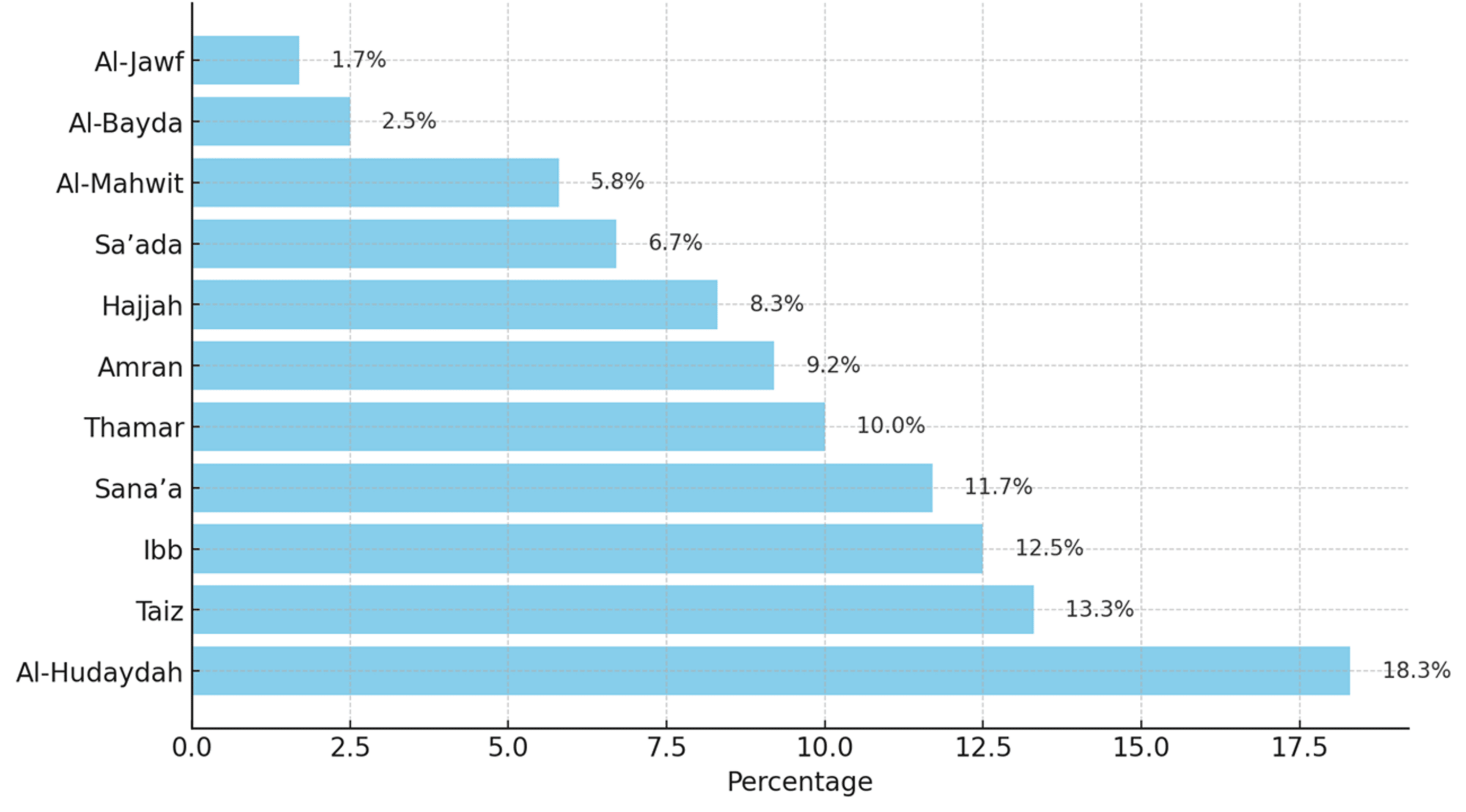
Geographic distribution of pediatric celiac disease cases.

The geographical distribution of CD cases revealed the highest prevalence in Al-Hudaydah (18.3%, n=22), followed by Taiz (13.3%, n=16) and Ibb (12.5%, n=15) (Figure 2).

Feeding practices and nutritional status

Feeding practices varied among children with CD. During the first six months, 41.7% (n=50) received mixed feeding, 32.5% (n=39) were bottle-fed, and 25.8% (n=31) were exclusively breastfed. Weaning occurred before six months in 46.7% (n=56) of children, with 53.3% (n=64) weaned at or after six months. Introduced foods included mixed foods (47.5%, n=57), dairy products (13.3%, n=16), and whole grains (11.7%, n=14) (Table 2). Nutritional assessment showed that 60.0% (n=72) of children were malnourished, defined as weight-for-age or height-for-age below -2 SD from WHO growth standards, while 40.0% (n=48) had normal nutrition (within ±2 SD). Vaccination data indicated that only 19.2% (n=23) were up to date, with 37.5% (n=45) partially vaccinated and 43.3% (n=52) unvaccinated, highlighting potential health vulnerabilities (Table 2).

**Table 2:** Distribution of nutritional status, vaccination history, and feeding practices among the study population (N=120) Descriptive statistics summarize the nutritional status, vaccination history, and feeding practices among the study population. Nutritional status is classified as Normal (within ±2 SD of WHO growth standards) or Malnutrition (below -2 SD of WHO standards).

Variable	Frequency	Percentage
Feeding pattern (first 6 months)		
Exclusive breastfeeding	31	25.8%
Bottle feeding	39	32.5%
Mixed feeding	50	41.7%
Weaning time		
<6 months	56	46.7%
≥6 months	64	53.3%
Weaning food		
Dairy products	16	13.3%
Whole grains	14	11.7%
Biscuits/cookies	12	10.0%
Legumes/eggs/meat	11	9.2%
Fruits and vegetables	10	8.3%
Mixed foods	57	47.5%
Nutritional status		
Normal (±2 SD)	48	40.0%
Malnutrition (	72	60.0%
Vaccination history		
Up to date	23	19.2%
Partial	45	37.5%
None	52	43.3%

Clinical manifestations

The most common gastrointestinal symptoms were chronic diarrhea (85.0%, n=102), abdominal pain (67.5%, n=81), and abdominal distention (65.0%, n=78). Other notable gastrointestinal symptoms included nausea and vomiting (55.0%, n=66) and loss of appetite (47.5%, n=57). Key extraintestinal symptoms encompassed pallor (81.7%, n=98), failure to thrive (70.8%, n=85), short stature (34.2%, n=41), headache (31.7%, n=38), irritability (30.8%, n=37), and rash (27.5%, n=33), reflecting CD’s systemic impact beyond the gastrointestinal tract. Less frequent conditions included vitamin deficiencies (14.2%, n=17) and a history of autoimmune diseases, such as hepatic disorders (2.5%, n=3) and thyroiditis (1.7%, n=2) (Table 3).

**Table 3:** Distribution of clinical manifestations among the study population (N=120) Descriptive statistics were applied to describe the clinical manifestations observed in the study population. Categorical variables are presented as frequencies and percentages to capture the prevalence of gastrointestinal and extraintestinal symptoms, as well as associated autoimmune conditions.

Variable	Frequency	Percentage
Gastrointestinal manifestations		
Chronic diarrhea	102	85.0%
Abdominal pain	81	67.5%
Abdominal distention	78	65.0%
Nausea and vomiting	66	55.0%
Loss of appetite	57	47.5%
Mouth ulcers	23	19.2%
Constipation	9	7.5%
Celiac crisis	4	3.3%
Extraintestinal manifestations		
Pallor	98	81.7%
Failure to thrive	85	70.8%
Short stature	41	34.2%
Headache	38	31.7%
Irritability	37	30.8%
Rash	33	27.5%
Dental enamel defects	32	26.7%
Chronic fatigue	21	17.5%
Developmental delay	21	17.5%
Vitamin deficiencies	17	14.2%
Arthritis	10	8.3%
Autoimmune disease history		
Hepatic disorders	3	2.5%
Thyroiditis	2	1.7%
Type 1 diabetes mellitus (T1DM)	1	0.8%

Associations between malnutrition and demographic variables

Table 4 presents the associations between nutritional status and demographic factors. Malnutrition showed significant associations with age, with higher rates in children under one year of age (79.2%, n=57 vs. 56.3%, n=27; p=0.019), and rural residency (73.6%, n=53 vs. 52.1%, n=25; p=0.015). Other factors linked to malnutrition included unprotected water sources (63.9%, n=46 vs. 43.8%, n=21; p=0.030), low family income (81.9%, n=59 vs. 54.2%, n=26; p=0.001), larger household size (≥5 persons, 75.0%, n=54 vs. 31.3%, n=15; p<0.001), none or low maternal education (80.6%, n=58 vs. 60.4%, n=29; p=0.016), positive family history of diarrhea (23.6%, n=17 vs. 2.1%, n=1; p=0.001), and incomplete vaccination (not up to date, 93.1%, n=67 vs. 62.5%, n=30; p<0.001). No significant association was found between malnutrition and gender (p=0.705) or contact with animals (p=0.053), suggesting these factors may not directly influence nutritional outcomes in this cohort (Table 4).

**Table 4:** Association between nutritional status and demographic variables The Chi-square test was used to assess the statistical significance of associations between nutritional status and each demographic or environmental variable. *Indicates a significant p-value (<0.05).

Variable	Malnutrition (n=72), n (%)	Normal (n=48), n (%)	Total (N=120), n (%)	Chi-square	p-value
Sex				0.144	0.705
Male	31 (43.1%)	19 (39.6%)	50 (41.7%)		
Female	41 (56.9%)	29 (60.4%)	70 (58.3%)		
Age				7.865	0.019*
<1 year	57 (79.2%)	27 (56.3%)	84 (70.0%)		
1–2 years	10 (13.9%)	11 (22.9%)	21 (17.5%)		
2–5 years	5 (6.9%)	10 (20.8%)	15 (12.5%)		
Residency				5.930	0.015*
Rural	53 (73.6%)	25 (52.1%)	78 (65.0%)		
Urban	19 (26.4%)	23 (47.9%)	42 (35.0%)		
Contact with animals				3.757	0.053
Yes	40 (55.6%)	18 (37.5%)	58 (48.3%)		
No	32 (44.4%)	30 (62.5%)	62 (51.7%)		
Water source				4.693	0.030*
Protected	26 (36.1%)	27 (56.3%)	53 (44.2%)		
Unprotected	46 (63.9%)	21 (43.8%)	67 (55.8%)		
Family income				11.449	0.001*
Low	59 (81.9%)	26 (54.2%)	85 (70.8%)		
Middle or high	13 (18.1%)	22 (45.8%)	35 (29.2%)		
Household size				17.398	<0.001*
<5 persons	18 (25.0%)	33 (68.8%)	51 (42.5%)		
≥5 persons	54 (75.0%)	15 (31.3%)	69 (57.5%)		
Maternal education				5.818	0.016*
None or low	58 (80.6%)	29 (60.4%)	87 (72.5%)		
Secondary or higher	14 (19.4%)	19 (39.6%)	33 (27.5%)		
Family history of diarrhea				12.932	0.001*
Positive	17 (23.6%)	1 (2.1%)	18 (15.0%)		
Negative	55 (76.4%)	47 (97.9%)	102 (85.0%)		
Vaccination history				12.158	<0.001*
Up to date	5 (6.9%)	18 (37.5%)	23 (19.2%)		
Partial	30 (41.7%)	15 (31.3%)	45 (37.5%)		
None	37 (51.4%)	15 (31.3%)	52 (43.3%)		

## Discussion

This study aimed to determine the prevalence, demographic characteristics, and clinical profiles of children diagnosed with CD admitted to the Al-Sabeen Hospital, Sana’a, Yemen. With a prevalence rate of 3.4% among admitted children, the findings reflect a higher-than-average occurrence of CD compared with global rates, which range from approximately 0.5% to 1% in the general population [14]. The 3.4% prevalence exceeds these estimates, possibly due to early gluten exposure (46.7% weaned before six months), as noted by Malekzadeh et al. [12], alongside genetic predispositions, dietary habits, and improved diagnostic awareness in Yemen, as suggested by Tack et al. [3]. Although direct data on the prevalence of CD in Yemen are limited, previous studies in high-risk populations with gastrointestinal symptoms have reported a prevalence of up to 9.2% [18]. A systematic review of CD in the Arab region similarly identified a prevalence of 0.2-1.2%, although higher rates were noted in neighboring countries, such as Saudi Arabia, where rates in children ranged from 3.1% in adolescents to 7.6% in specific risk groups [10,19]. The increased rate of diagnosis among younger children, with 70% of cases identified within 12 months, underscores the importance of early screening and intervention in high-risk children. This pattern is consistent with prior research, which noted that early childhood is often the period when CD symptoms become more evident [20].

A significant proportion of CD cases in this study (65%) were from rural areas, with a high percentage of patients from low-income households (70.8%) and mothers with minimal education (72.5%). These socioeconomic factors are consistent with the broader conditions in Yemen, where conflict has intensified poverty and limited access to health care [21,22]. Studies have shown that socioeconomic status is a critical determinant of accessing healthcare, especially for identifying chronic conditions such as CD, where early intervention can be lifesaving [23]. Rural predominance may indicate limited healthcare access and possible underdiagnosis in rural areas, highlighting the need for community health education to improve CD awareness. The lack of a significant association between malnutrition and sex (p=0.705) or animal contact (p=0.053) suggests that these factors may not directly influence nutritional outcomes in this cohort, although the trend toward significance for animal contact (48.3% exposure) and unprotected water sources (55.8%, p=0.030) points to environmental exposure that warrants further investigation in larger studies.

Feeding practices among the children in this study revealed early weaning, with 46.7% weaned before six months and 41.7% receiving mixed feeding. Although common, these practices may increase the risk of developing CD in genetically predisposed children, especially when gluten is introduced early. Previous studies have suggested that early gluten exposure can trigger symptoms in children with CD [3,12]. The high prevalence of malnutrition (60%) in this cohort, consistent with the findings of Al-Waleedi and Bin-Gouth [24], reflects the compounded effects of impaired nutrient absorption due to CD and food insecurity in conflict zones. These findings underscore the need for community education on breastfeeding, optimal weaning times, and the safe introduction of solid foods, particularly in areas affected by conflict, where healthcare guidance may be scarce.

Vaccination coverage among children in this study was low, with 43.3% of children unvaccinated, reflecting broader immunization challenges in Yemen. Political instability and economic hardship limit access to essential vaccines, especially in rural areas [25,26]. The strong association between incomplete vaccination and malnutrition (93.1% in malnourished vs. 62.5% in normal, p<0.001) suggests that children with CD may face heightened health risks due to compromised immune systems and nutritional deficits. To address this, immunization programs should prioritize high-risk populations, including those with chronic conditions, such as CD, to reduce susceptibility to preventable diseases.

Significant associations were found between malnutrition and several factors, including younger age, rural residency, unprotected water sources, low family income, larger family size, non-education or low maternal education, positive family history of diarrhea, and incomplete vaccination history. These findings underscore the multifactorial nature of malnutrition in CD patients and highlight the need for comprehensive public health strategies to address these issues [27-29]. For instance, the link with unprotected water sources (63.9%, p=0.030) may reflect poor sanitation, exacerbating diarrhea and nutrient loss, a known risk in Yemen [20]. Similarly, low maternal education (80.6%, p=0.016) aligns with studies showing its impact on child health outcomes in low-resource settings [29].

The findings underscore the need for targeted public health initiatives to address CD in conflict-affected areas. First, community-based screening programs, particularly in high-risk groups, can improve early detection rates and encourage timely interventions. Educational campaigns focusing on CD awareness, symptom recognition, and nutritional management can equip health care providers and families to recognize CD symptoms earlier. Mobile health units or community health workers can deliver these services to remote areas, thereby increasing healthcare accessibility. Moreover, strengthening partnerships with local NGOs to distribute gluten-free food options and essential vaccines could address the key health gaps intensified by the ongoing conflict. Nutritional support programs, including education on CD-compatible foods and access to nutrient-dense, gluten-free options, could mitigate the high malnutrition rates observed by building on models proposed for other conflict zones [24].

Limitations and future research

The cross-sectional design of this study limits causal inference, as noted by Ludvigsson and Murray [1], preventing the establishment of temporal relationships between CD and associated sociodemographic or environmental factors. For instance, while associations between malnutrition and factors such as low family income or unprotected water sources were identified, the retrospective nature of the data could not confirm whether these factors preceded or resulted from CD. Additionally, the single-center focus at Al-Sabeen Hospital may not fully represent Yemen’s diverse population, a challenge highlighted in regional studies [10]. This restriction potentially underestimates the prevalence variations across urban and rural settings beyond Sana’a, where healthcare access and diagnostic capabilities differ. Recall bias is another concern, as data on feeding practices, vaccination history, and family history relied on parental reporting, which may be subject to inaccuracies, especially in conflict-affected regions with disrupted record-keeping.

The absence of genetic testing, such as human leukocyte antigen (HLA)-DQ2 and HLA-DQ8 typing, limits insight into the genetic susceptibility of Yemeni children to CD. Given the potential influence of genetic factors on prevalence, as suggested by Malekzadeh et al. [12], this omission restricts the ability of the study to contextualize findings within the broader epidemiology of CD in the Middle East. Furthermore, the study did not assess the long-term outcomes of CD management, such as adherence to a gluten-free diet or resolution of malnutrition, owing to its retrospective scope.

Future research should include multicenter and longitudinal studies, as recommended by El-Metwally et al. [10], to enhance generalizability and to explore causality. Incorporating genetic testing (e.g., HLA-DQ2 and HLA-DQ8 typing) would further clarify genetic susceptibility in the region, as suggested by Malekzadeh et al. [12], potentially explaining the higher prevalence observed (3.4%) compared to global averages [14]. Expanding data collection to include prospective follow-up could assess the impact of interventions such as gluten-free diets and nutritional support on clinical outcomes, thus addressing gaps in management efficacy. Additionally, investigating environmental factors such as water quality and animal contact in greater detail with larger samples could elucidate their role in CD presentation and malnutrition, building on the trends noted (p=0.030 and p=0.053, respectively). Such studies would provide a more comprehensive understanding of CD in Yemen and inform targeted public health strategies in similar resource-limited, conflict-affected settings.

## Conclusions

This study revealed a significant prevalence of CD among children admitted to Al-Sabeen Hospital in Sana’a, Yemen, with 3.4% of 3,570 admissions diagnosed over a five-year period, alongside a substantial proportion affected by malnutrition. The findings highlight a higher-than-expected burden of CD in this conflict-affected region, with female predominance, a majority of diagnoses in children under 12 months of age, and strong associations between malnutrition and socioeconomic factors such as low family income, rural residency, and incomplete vaccination. 

For patients with tTG-IgA levels below 10 times the upper limit of normal, we recommend biopsy confirmation, as applied in this study, using the ESPGHAN online diagnostic tool to ensure guideline-aligned diagnoses. These results underscore the need for enhanced screening, nutritional support, and public health interventions tailored to resource-limited settings, such as Yemen, where early detection and management of CD can mitigate its severe clinical consequences.

## Figures and Tables

**Figure 3:**
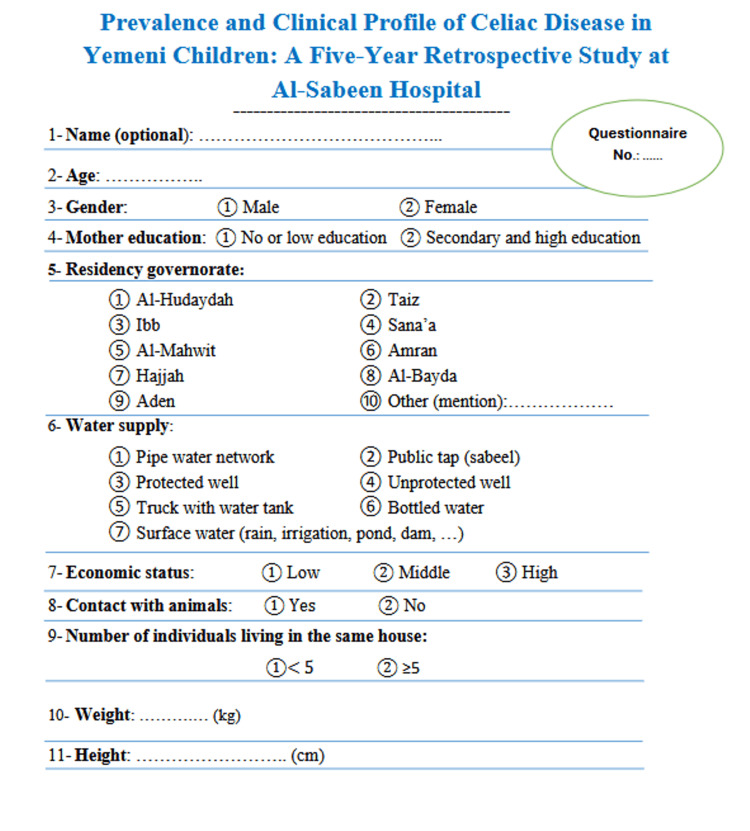
Questionnaire page 1

**Figure 4:**
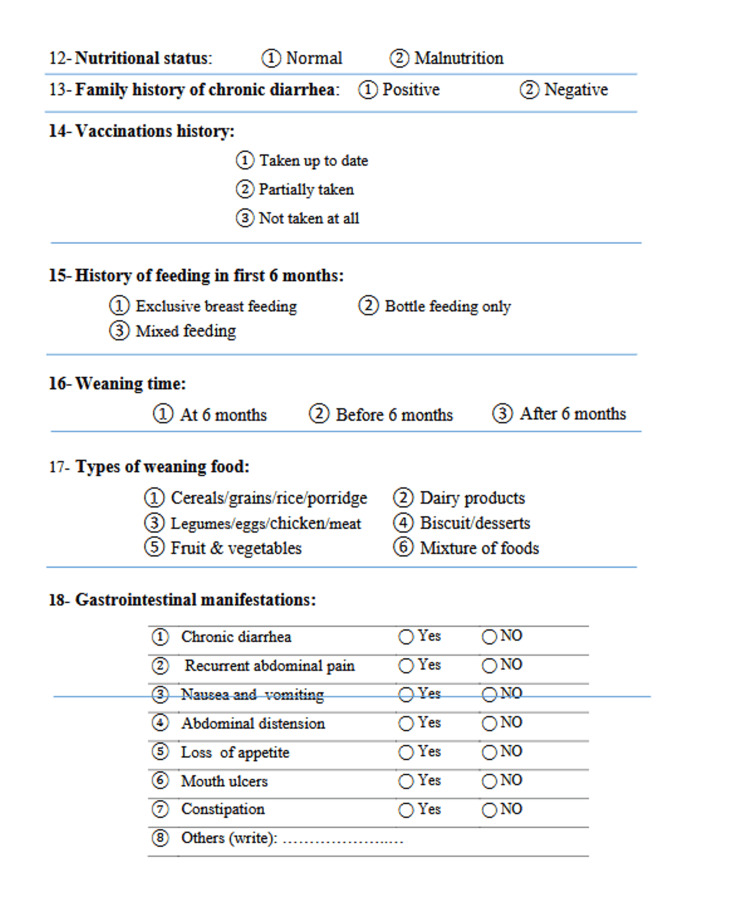
Questionnaire page 2

**Figure 5:**
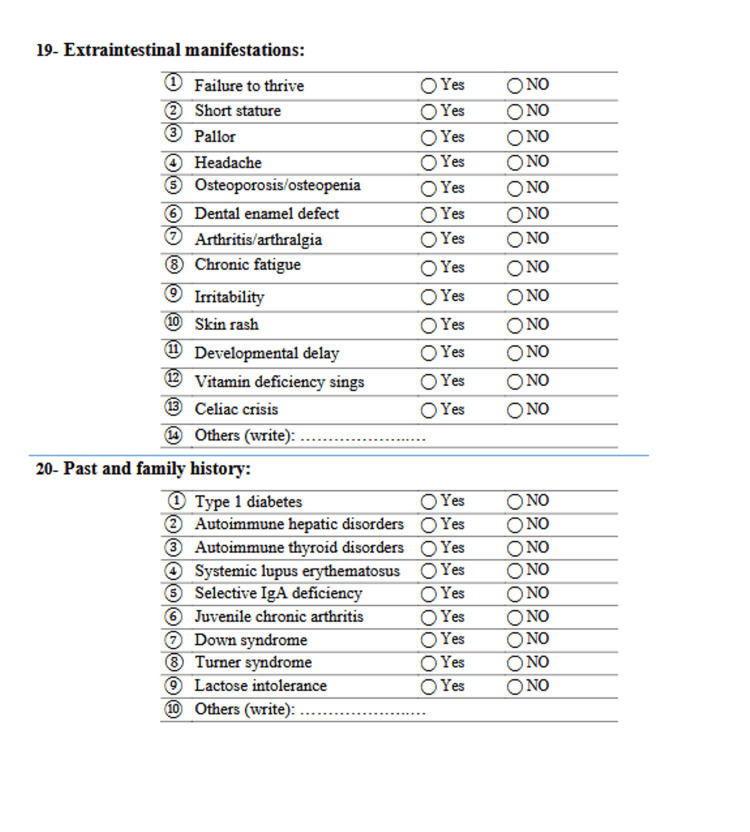
Questionnaire page 3
